# Thymoma: A case report and update

**DOI:** 10.7196/SARJ.2019.v25i1.229

**Published:** 2019-04-12

**Authors:** N Mathiselvan, E M Irusen, C F N Koegelenberg

**Affiliations:** Division of Pulmonology, Department of Medicine, Stellenbosch University and Tygerberg Academic Hospital, Cape Town, South Africa

**Keywords:** Thymoma, mediastinal mass

## Abstract

Thymomas are slow growing, evolve with metastasis and commonly present as ipsilateral pleural involvement along with the primary tumour of the mediastinum. Early-stage recognition and treatment with surgical resection and postoperative chemoradiotherapy may offer a better 10-year survival rate.

## Background

Thymoma is a malignant neoplasm that arises from the epithelial cells of the thymus gland, a lymphoid organ situated in the anterior mediastinum. It is one of the most commonly reported primary tumours of the anterior mediastinum.^[1,2]^ The peak incidence is found in patients between 50 and 60 years of age. Thymomas are slow-growing neoplasms that may show aggressive characteristics involving invasion of adjacent mediastinal structures and metastasis to the pleura and pericardium.^[3]^

## Case

**Fig. 1:**
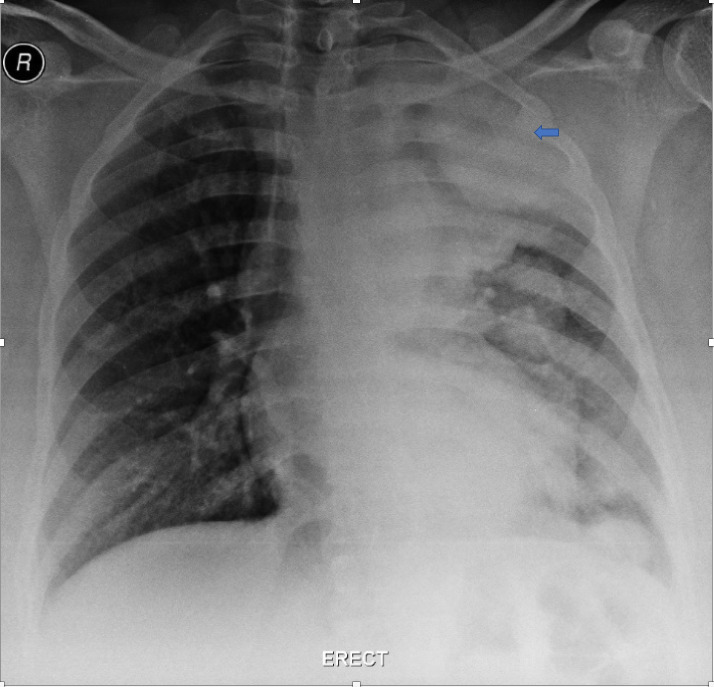
The chest radiograph showed a homogeneous mass lesion in the left upper zone, with multiple pleural-based mass lesions.

A 40-year-old female patient was referred to our service from a district hospital. She complained of left-sided infraclavicular pain for 4 months. A chest radiograph revealed a homogeneous opacity in the left upper zone, with multiple pleural-based mass lesions (Fig. 1).

**Fig. 2:**
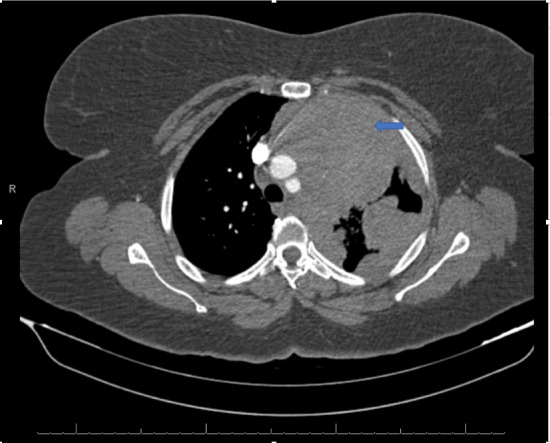
The computed tomography scan of the chest showed a large lobulated soft-tissue anterior mediastinal mass, with multiple nodular pleural-based mass lesions on the left side.

A subsequent computed tomography (CT) scan revealed a large lobulated soft-tissue anterior mediastinal mass (measuring 60 Hounsfield units (HU)), with multiple pleural nodular masses (Fig. 2).

**Fig. 3:**
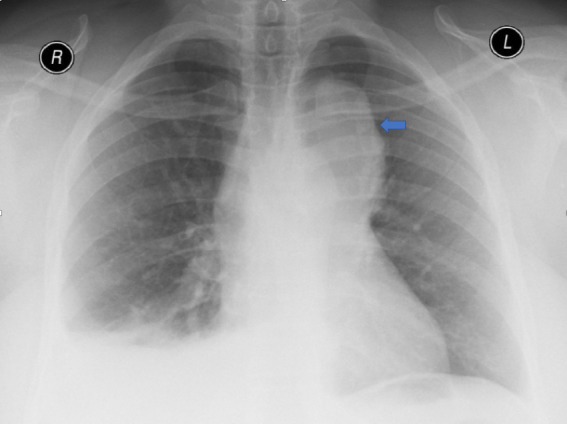
The chest radiograph, taken 4 years prior to the current presentation, revealed a mass lesion in the left mid to upper zone. A clear hilum overlay sign was present and the descending aorta was clearly visible, suggesting an anterior mediastinal mass.

A soft-tissue mass of 3 cm in diameter was also seen in the lateral limb of the left adrenal gland. The patient’s previous images captured on the local healthcare system’s radiology archive were reviewed and it was noted that a chest radiograph performed 4 years earlier also showed the mass lesion in the anterior mediastinum (Fig. 3). A chest CT scan was planned at the time, but the patient failed to return to our institution.

An ultrasound-guided Tru-cut needle biopsy of the mass lesion was performed. Histopathological examination revealed epithelioid cells growing in sheets, composed of bland oval cells with oval nuclei that were euchromatic without nucleoli. On further immunohistochemical staining, the cytokeratin marker MNF116 was positive in epithelioid cells and CD3 and terminal deoxynucleotidyl transferase were positive in the lymphoid component, which confirmed the diagnosis of a type B2 thymoma. She was thus staged as IVB thymoma B2 based on the Masaoka-Koga staging system.^[4]^

## Discussion

Thymic epithelial neoplasms are rare malignant tumours that arise from the thymic gland. These neoplasms include thymoma, thymic carcinoma and neuroendocrine tumours of thymic origin, of which thymoma is the most common.^[5]^ The incidence of thymoma increases with age and peaks in the fifth decade of life, with men and women equally affected. Approximately 50% of nearly all types are detected incidentally in asymptomatic patients on chest imaging examination.^[6]^ When symptoms are present, they are usually due to local effects of the malignancy, including compression and invasion of adjacent structures, which can result in dysphagia, diaphragmatic paralysis or superior vena cava syndrome. It has been reported that nearly a third of patients report chest pain, dyspnoea or cough. Patients may also present with paraneoplastic syndromes owing to the frequent association of immune-mediated systemic diseases, of which myasthenia gravis is the most common (seen in 30% - 50% of patients with thymoma). Hypogammaglobulinaemia and pure red cell aplasia are the other significant paraneoplastic syndromes associated with thymoma.^[7]^

Thymomas are composed of neoplastic epithelial cells and lymphocytes that are non-neoplastic in nature and exhibit marked variations in histology. According to the recent histological classification system by the World Health Organization consensus committee, thymoma is classified into five subtypes: A, AB, B1, B2 and B3. The committee also noted that several subtypes can coexist in the same tumour, which poses a challenge to classification; however, this has no clinical implication.^[8]^

The clinical course and decisions are based on the stage of the disease. Staging of thymoma has conventionally been based on either the Masaoka or the Masaoka-Koga staging systems, with the latter recently being recommended by the International Thymic Malignancy Interest Group.

A CT scan is the modality of choice for the evaluation of thymoma. It usually presents as an anterior mediastinal mass of 1 - 10 cm, round in shape and with well-defined margins and occasional calcification. Nearly a third of all thymomas are potentially invasive, infiltrating through the capsule into the nearby structures. Advanced-stage thymomas exhibit pleural spread or ‘drop’ metastases, which show up as one or multiple pleural nodules and are usually ipsilateral to the primary tumour.^[6]^

The 10-year overall survival rates for stage I, II, III, IVA and IVB thymomas are 84%, 83%, 70% ,42% and 53%, respectively. The treatment strategy is based on tumour resectability; completeness of resection is the most important predictor of outcome. Postoperative radiotherapy is recommended only for incompletely resected thymomas, which should be administered by the three-dimensional conformal technique to reduce damage to surrounding normal tissue and coupled with the use of intensity-modulated radiotherapy. Induction chemotherapy is recommended for locally advanced thymomas, followed by an evaluation for surgery; postoperative radiotherapy can be considered after resection of the primary tumour. In those with solitary metastasis or ipsilateral pleural metastases, treatment options include induction chemotherapy or surgery. In the case of unresectable disease, radiotherapy with or without chemotherapy is recommended. Although six different combination regimens are available in the National Comprehensive Cancer Network algorithm of 2018, a combination of cisplatin, doxorubicin and cyclophosphamide is regarded as the regimen of choice for thymoma.^[9]^ After primary treatment of resectable thymomas, surveillance for recurrence should include six-monthly chest CT scans for 2 years and then annually for 10 years. Given the current treatment recommendation, we administered multiagent induction chemotherapy for our patient (cyclophosphamide, doxorubicin and cisplatin) to relieve tumour-related symptoms.
